# Inhibition of Uterine Sarcoma Cell Growth through Suppression of Endogenous Tyrosine Kinase B Signaling

**DOI:** 10.1371/journal.pone.0041049

**Published:** 2012-07-23

**Authors:** Kenichi Makino, Kazuhiro Kawamura, Wataru Sato, Nanami Kawamura, Toshio Fujimoto, Yukihiro Terada

**Affiliations:** 1 Department of Obstetrics and Gynecology, Akita University School of Medicine, Akita, Japan; 2 Dermatology and Plastic Surgery, Akita University School of Medicine, Akita, Japan; University of Louisville, United States of America

## Abstract

Uterine leiomyosarcoma is an aggressive tumor typically found at advanced stages due to difficulties with early diagnosis. Because uterine leiomyosarcoma is resistant to conventional radiation and chemotherapy, the development of more potent medical therapeutics is anticipated. Using quantitative real-time RT-PCR and immunostaining, we found the expression of brain-derived neurotrophic factor (BDNF) and neurotropin-4/5, together with their receptor, tyrosine kinase B (TrkB), in different uterine sarcoma cell lines and primary tumor samples from uterine leiomyosarcoma patients. We noted that levels of BDNF were more abundant than those of neurotropin-4/5. Moreover, the expression of TrkB and its ligands was elevated in a multidrug-resistant cell line and samples obtained from patients with leiomyosarcoma. In cultured uterine sarcoma cells, inhibition of endogenous TrkB signaling by treatment with either the soluble TrkB ectodomain or the Trk receptor inhibitor, K252a, suppressed cell proliferation and increased apoptosis based on cell viability and proliferation, *in situ* terminal deoxynucleotidyl transferase-mediated 2′-deoxyuridine 5′-triphosphate nick end-labeling and caspase-3/7 assays, whereas an inactive plasma membrane nonpermeable K252b was ineffective. Correspondingly, treatment with exogenous BDNF increased cell proliferation. In *in vivo* studies in athymic nude mice bearing multidrug-resistant uterine sarcoma cell tumors, we demonstrate suppression of tumor growth by treatment with K252a, but not K252b, as reflected by decreased cell proliferation and increased levels of apoptosis and caspase-3/7 activities without obvious side effects. Our findings indicated that endogenous signaling of the TrkB pathway contributed to uterine sarcoma cell growth, and inhibition of TrkB signaling in these tumors could provide a novel medical therapy for patients with uterine sarcomas.

## Introduction

Leiomyosarcoma is most common subtype among uterine sarcomas. The most effective treatment for this disease is a complete resection of the primary lesion at an early stage. However, a differential diagnosis between early stage uterine leiomyosarcomas and myomas is difficult. In fact, a uterine leiomyosarcoma diagnosis is often made after surgery for benign uterine myomas [1], [2]. If surgical remission can not achieve, the clinical outcome is poor as both radiation therapy [3], [4] and chemotherapies [5]–[11] have little to no effect [12]. In spite of the aggressive character and poor clinical outcome of uterine leiomyosarcomas [13], standard therapies have not been established due to difficulties with early diagnosis and drug-resistant phenotypes. Thus, the development of new therapeutic approaches is necessary to treat this disease.

Brain-derived neurotrophic factor (BDNF) belongs to the neurotrophin family, and binds to the receptor tyrosine kinase B (TrkB) and the pan-neurotrophin receptor p75 (p75NTR) with high and low affinities, respectively [14], [15]. BDNF has been characterized primarily through its induction of TrkB signaling in central nervous system (CNS) development, neuronal survival and synaptic plasticity [16]. Trk was first identified as an oncogene [17] and its role in neuroblastomas has been well-characterized [18]. Specifically, TrkB activation by BDNF promotes cell growth and induces drug-resistant neuroblastoma phenotypes [19]–[22]. Recently, several lines of evidence for the involvement of TrkB signaling in non-neurogenic cancers, including breast, ovarian, and Wilms’ tumor, have been reported [23]. These data suggest a potential role for BDNF/TrkB signaling in malignant tumor growth.

The placenta is a fast-growing organ that displays some tumor-like properties, e.g., high rates of trophoblast cell proliferation and invasion. We demonstrated that BDNF promotes proliferation and survival of trophectoderm cells before implantation [24] and trophoblast cell growth and survival during placental development after implantation in pregnancy [25]. In addition to the expression of BDNF and TrkB in embryo, their expression was also detected in the uterine smooth muscle cells (unpublished data). Together, these results prompted us to investigate the endogenous roles of BDNF/TrkB signaling in the malignant uterine smooth muscle tumor, leiomyosarcoma. Here, we showed the expression of TrkB and its ligands in human uterine leiomyosarcoma, and demonstrated an endogenous regulatory effect of TrkB on *in vitro* cell growth and survival using the soluble ectodomain of TrkB and a pan-Trk receptor inhibitor. We also demonstrated that the levels of TrkB and BDNF transcripts were elevated in samples obtained from patients with leiomyosarcoma as compared with those of uterine myometrium and leiomyoma. Furthermore, we showed that a Trk inhibitor suppressed tumor growth in athymic nude mice bearing uterine sarcoma cell tumors.

## Materials and Methods

### Cell Lines

The human uterine sarcoma cell lines, MES-SA and MES-SA/Dx5, and human uterine leiomyosarcoma cell line, SKN were purchased from the American Type Culture Collection (Manassas, VA, USA) and the Japan Health Science Foundation (Osaka, Japan), respectively. MES-SA/Dx5 is a multidrug-resistant variant of MES-SA developed by Harker *et al*. [26]. MES-SA and MES-SA/Dx5 cells were maintained in McCoy’s 5A medium (GIBCO BRL, Palo Alto, CA, USA), whereas SKN cells were maintained in Ham’s F12 medium, supplemented with 10% fetal bovine serum (FBS; Invitrogen, Carlsbad, CA, USA), penicillin (100 U/ml), and streptomycin (100 µg/ml) at 37°C in 5% CO_2_.

### RT-PCR and Immunostaining

To study the expression of tyrosine receptor kinase (Trk) family and thier ligands, conventional RT-PCR was performed. Total RNA was extracted from cells and tissues using an RNeasy Mini kit (Qiagen, Hilden, Germany). Uterine leiomyosarcoma tissue sample was obtained from patients at the Department of Obstetrics and Gynecology, Akita University, during surgical resection with ethical committee approval and informed consent from patients. PCR primers for TrkB, p75NTR, BDNF, NT4/5 and β-actin were used as described [25], [27], [28]. As a negative control, experiments were performed without template DNA. To confirm identity, bands of each PCR product were eluted from the agarose gel using the QIAquick gel extraction kit (Qiagen), ligated into the pDrive Cloning vector (Qiagen), and cloned in accordance with standard protocols. Plasmid DNA was recovered using Quantum Prep Plasmid Miniprep kit (Bio-Rad Laboratories, Inc., Hercules, CA, USA), cycle sequenced, and analyzed in a ABI 3130 Genetic Analyzer (Life Technologies Japan, Tokyo, Japan) using T7 or SP6 site-specific primers.

Immunofluorescent detection of TrkB and their ligands in MES-SA/Dx5 cells was performed as described [24], [27]. TrkB, BDNF, or NT-4/5 antigens were detected by chicken anti-TrkB polyclonal antibodies recognizing the full-length TrkB (Promega, Madison, WI, USA), rabbit anti-BDNF polyclonal antibodies (Chemicon, Temecula, CA, USA) or rabbit anti-NT4 polyclonal antibody (Chemicon), respectively, at 1∶500 dilutions.

### Quantitative Real-time RT-PCR

Total RNA was extracted from cell lines and frozen surgical specimens of human myometrium (normal uterine smooth muscle) and uterine leiomyoma (uterine benign tumor) using RNeasy Mini kit (Qiagen). Due to insufficient number of frozen samples of uterine leiomyosarcoma, total RNA was extracted from formalin-fixed parafine-embeded (FFPE) samples using RNeasy FFPE kit (Qiagen). After reverse transcription of 1 µg tRNA of each samples by Prime Script RT reagent kit (TaKaRa, Shiga, Japan), real-time PCR was performed using LightCycler 480 system with SYBR Green I Master mix (Roche Applied Science, Indianapolis, IN, USA). Primers for β-actin, TrkB, BDNF, NT-4/5, and galectin-1 were as follows:

β-actin: sense, 5′-CCACACTGTGCCCATCTACG-3′; antisense, 5′-AGGATCTTCATGAGGTAGTCAGTCAG-3′; TrkB: sense, 5′-CTGAAGGATGCCAGTGACAA-3′; antisense, 5′-ATGTGCTCATGCTGGAGGTT-3′; BDNF: sense, 5′-GCCAGGACAGAGCTGACAA-3′; antisense, 5′-TCCATGAACAGACAGGATGG-3′; NT4/5: sense, 5′-TGGATTCGAATTGACACTGC-3′; antisense, 5′-CCTGAGGTCTCTCAGCATCC-3′; galectin-1: sense, 5′-TGCAACAGCAAGGACGGC-3′; antisense, 5′-CACCTCTGCAACACTTCCA-5′. In all reactions, HotStartTaq DNA polymerase was activated by an initial denaturation at 95°C for 5 min, followed by 45 cycles of denaturation at 95°C for 10 sec and annealing at/elongation at β-actin, 64°C; TrkB, BDNF and NT4/5, 60°C; Galectin-1, 61°C for 10 sec and elongation at 72°C for 10 sec. Fluorescent signals were monitored at the end of the elongation phase for each cycle. Data were normalized based on β-actin transcript levels. Human tissue samples were obtained from patients at the Department of Obstetrics and Gynecology, Akita University with ethical committee approval and informed consent from patients.

### *In vitro* Analyses of Cell Proliferation and Apoptosis

To examine roles of endogenous Trk ligands in cell proliferation and apoptosis, MES-SA, MES-SA/Dx5 and SKN cells were seeded in 96-well culture plates at a density of 300 cells/well. Cells were incubated overnight before beginning a 48 h culture in serum-free medium with or without different doses of the following compounds: the TrkB soluble ectodomain (R&D systems, Minneapolis, MN, USA); the pan-specific Trk inhibitor, K252a [Calbiochem, La Jolla, CA, USA, [29]]; or its analog, the inactive, plasma membrane impermeable K252b [Calbiochem, [30]]. The doses of K252a and K252b chosen for these experiments were based on previous studies [31], [32].

Cell proliferation was determined using WST1 (Roche). Absorbance at 450 nm was measured by a plate reader (BioRad, Hercules, CA, USA). To measure cellular apoptosis, quantitative caspase-3/7 enzyme assay was performed as described [33]. Apoptosis in cells was also assayed by detecting DNA fragmentation using in situ terminal deoxynucleotidyl transferase-mediated dUDP nick end-labeling (TUNEL) [Roche, [34]] For the latter test, the cells and tissues were treated with DNase I as positive controls, and were incubated without the labeling enzymes as negative controls (data not shown).

To determine the effects of BDNF on cell proliferation, MES-SA and MES-SA/Dx5 cells were first plated in 96-well culture plates and incubated overnight. Then, they were cultured with or without different doses of BDNF (R&D systems) for 48 h in serum-free medium. At the end of cultures, cell proliferation was measured using the WST1 assay.

**Figure 1:**
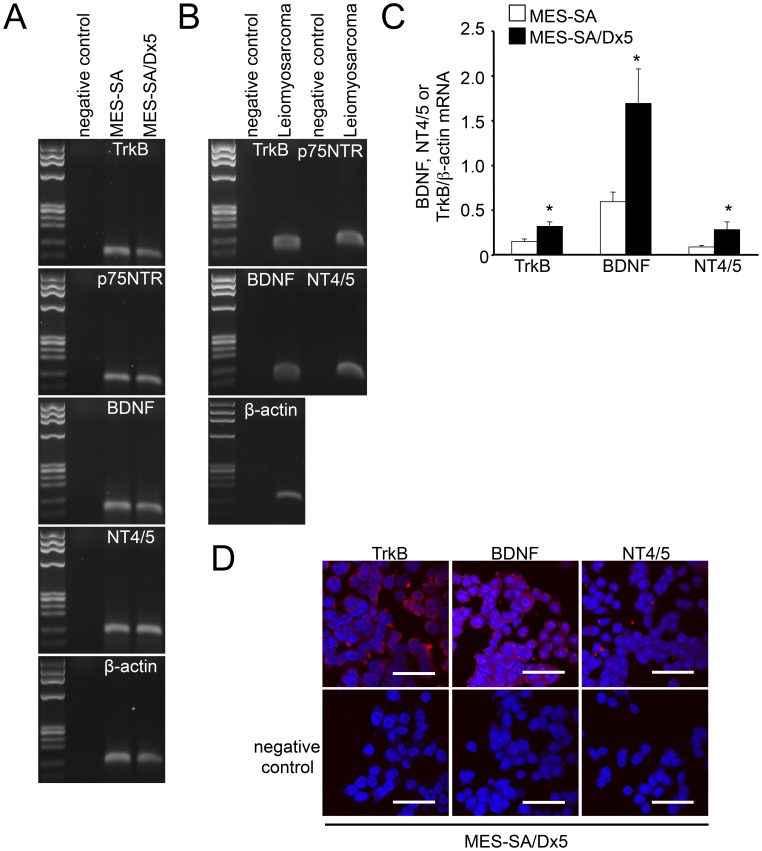
Expression of TrkB, p75NTR, BDNF and NT4/5 in uterine sarcoma cell lines and human uterine leiomyosarcoma tissues. (A) Messenger RNA expression of TrkB, p75NTR and their ligands was detected by RT-PCR in the uterine sarcoma cell lines, MES-SA and MES-SA/Dx5. As loading controls, the levels of β-actin were also assessed. The negative control lacked template DNA. (B) Messenger RNA expression of TrkB, p75NTR, and their ligands, BDNF and NT4/5, in surgical specimens from human uterine leiomyosarcomas was detected by RT-PCR. As loading controls, β-actin mRNA levels were assessed. (C) Expression levels of TrkB, BDNF and NT4/5 in uterine sarcoma cell lines were quantified by real-time RT-PCR. The expression level of each transcript was standardized using levels of β-actin transcripts in the same samples (n = 4). *Columns*, mean; *bars*, SE. *, *P*<0.05 vs. MES-SA. (D) Immunohistochemical detection of TrkB, NT4/5 and BDNF proteins. Target proteins (red signal) were detected in MES-SA/Dx5 cells (Upper panels). Lower panels depict negative controls. Nuclei (blue) were stained with Hoechest 33342. (Scale bars, 50 µm).

**Figure 2:**
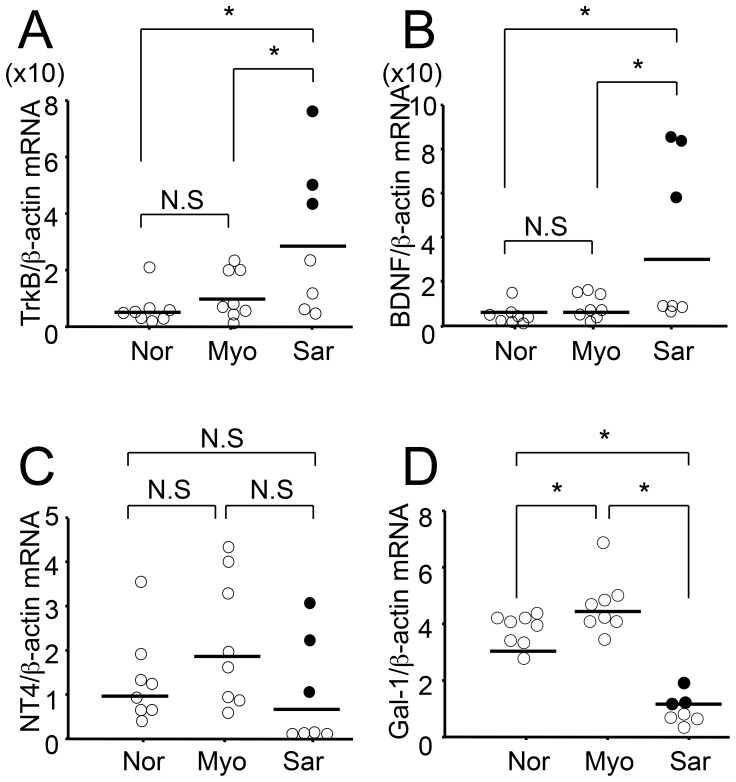
Expression levels of TrkB, BDNF, NT4/5 and galectin-1 in human uterine myometrium, leiomyoma and leiomyosarcoma. To compare the expression levels of TrkB (A), BDNF (B), NT4/5 (C) and galectin-1 (D) in human uterine myometrium (n = 8), leiomyoma (n = 8) and leiomyosarcoma (n = 7), quantitative real-time RT-PCR was performed. The expression level of each transcript was normalized using transcript levels of β-actin in the same samples. Nor, normal myometrium; Myo, leiomyoma; Sar, leiomyosarcoma. Same corresponding samples were indicated as closed circles. Results were represented by scatter diagrams. Horizontal lines indicate medians. *, *P*<0.05. N.S, not significant.

**Figure 3:**
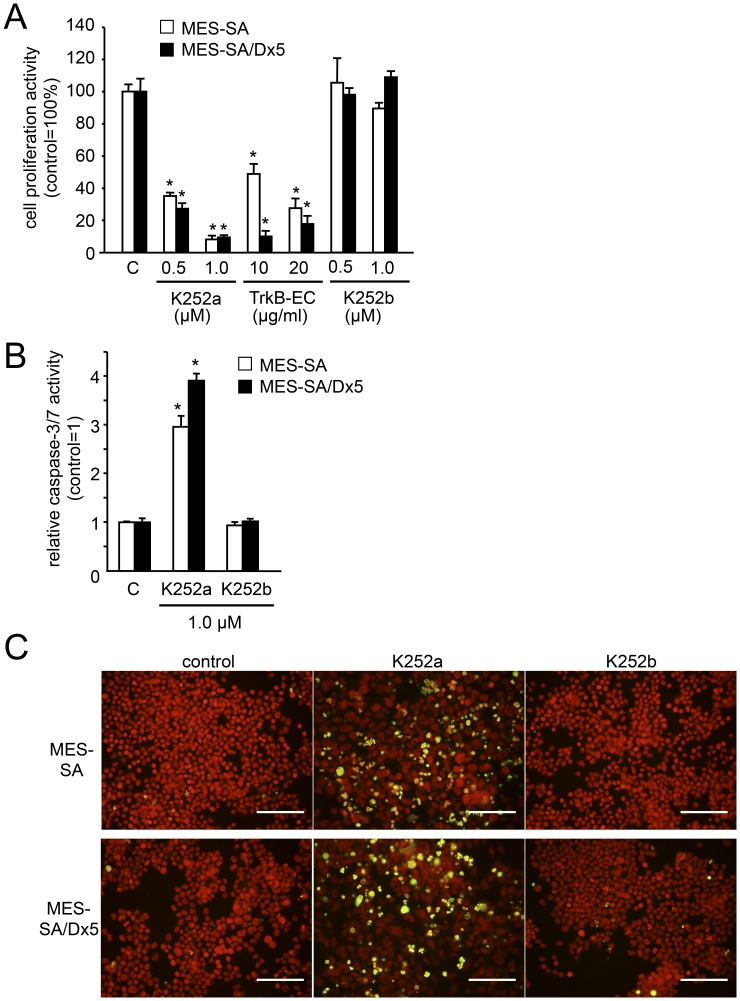
Roles of endogenous TrkB ligands in *in vitro* growth of uteine sarcoma cells. To determine the effects of endogenous TrkB ligands on cell proliferation (A) and survival (B), uterine sarcoma cells (MES-SA/Dx5 cells) were cultured in medium alone (control, C), or with different doses of the TrkB ectodomain (TrkB EC), the pan-Trk inhibitor, K252a, or its inactive analogue, K252b. Cell proliferation activity was determined using the cell proliferation reagent WST1 after 48 h of culture, and cellular apoptosis was quantified using the caspase-3/7 assay after 8 h of culture (n = 3). *Columns*, mean; *bars*, SE. *, *P*<0.05 vs. control. (C) DNA fragmentation was detected by *in situ* TUNEL staining. Nucleic acids are stained with propidium iodide (red). Representative images were obtained from MES-SA (upper panels) and MES-SA/Dx5 (lower panels) cells after treatment ± K252a (1 µM) or K252b (1 µM). The number of apoptotic cells (green) was increased in the cells with K252a treatment. (Scale bars, 100 µm).

**Figure 4:**
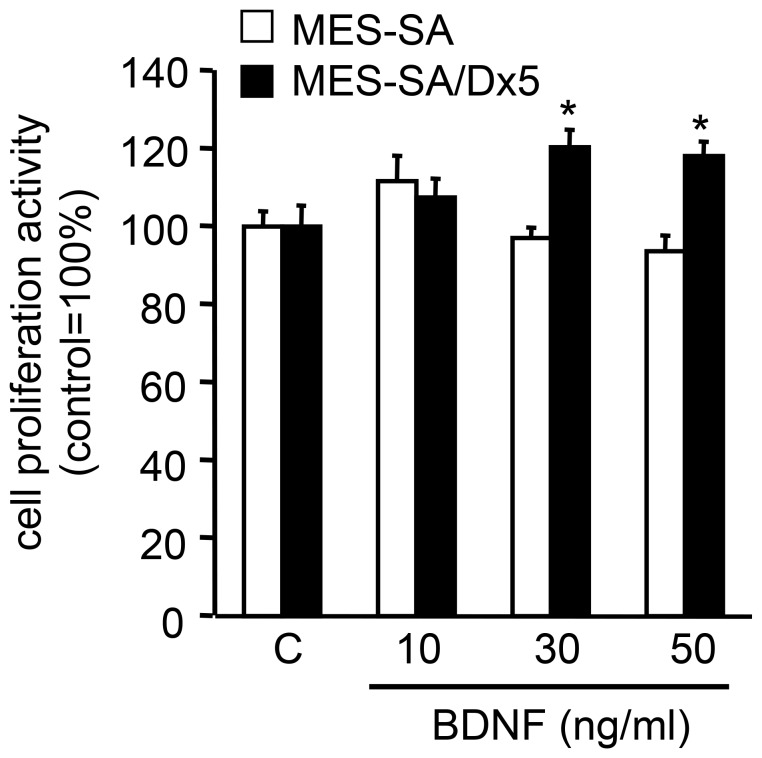
Promotion of cell proliferation in uterine sarcoma cells. MES-SA and MES-SA/Dx5 cells were cultured in medium alone or with different doses of BDNF. After incubation for 48 h, cell proliferation activity was measured by WST1 (n = 3). *Columns*, mean; *bars*, SE. *, *P*<0.05 vs. control.

**Figure 5:**
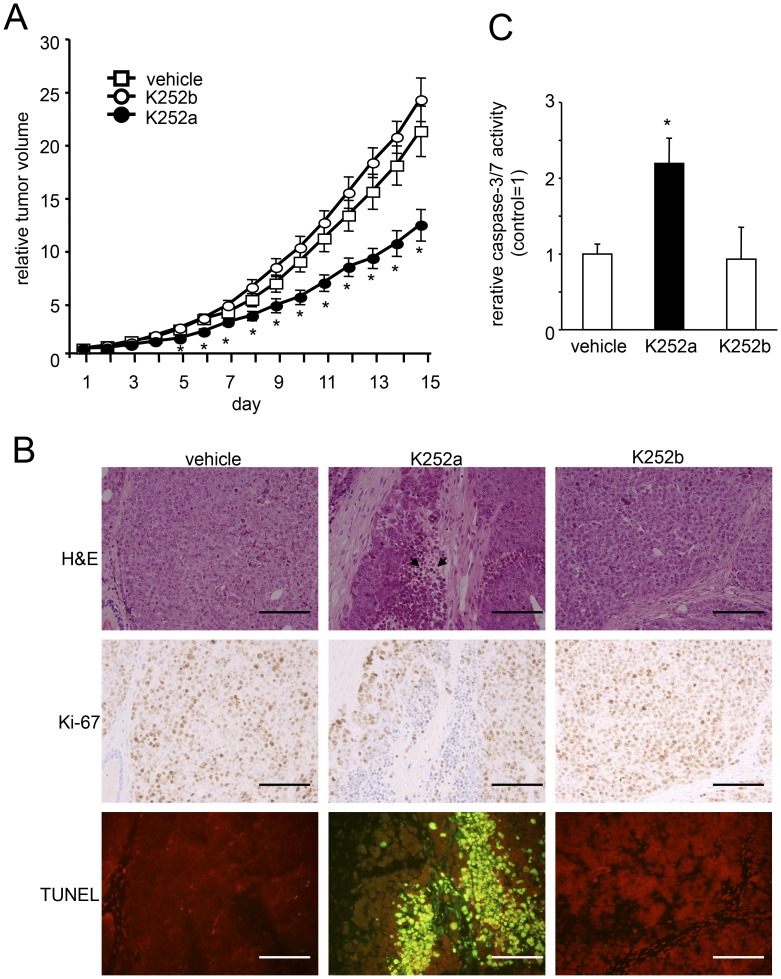
Effect of Trk receptor inhibitor on *in vivo* tumor growth of MES-SA/Dx5 xenografts in athymic nude mice. (A) Time course of tumor growth in mice treated every 3 days with vehicle alone, K252a, or K252b (1 mg/kg, n = 6). Tumor volume on the first day of treatment was expressed as a relative tumor volume of 1. *Points*, mean; *bars*, SE. *, *P*<0.05 vs. control. (B) Histological characterization of cell proliferation and apoptosis in tumors. In H&E staining of K252a-treated tumor tissue, the number of mitotic cells was decreased and cells with chromatin condensation (arrowhead) was increased. K252a treatment decreased Ki-67 signals and increased the number of TUNEL stained (green) nuclei. (Scale bars, 100 µm). (C) Caspase-3/7 activities in tumors. Samples were obtained from the mice bearing MES-SA/Dx5 tumors after 15 days of treatment. Data were standardized by protein concentrations, expressed as the fold increase relative to controls (vehicle alone), and normalized to 1. *Columns*, mean; *bars*, SE. *, *P*<0.05 vs. control group.

### *In vivo* Studies

To explore the roles of endogenous Trk ligands during *in vivo* tumorigenesis of uterine sarcomas, the antitumor activity of K252a was assessed in 4-week-old female athymic nude mice (BALB/c nu/nu) (SLC Japan, Tokyo, Japan) bearing MES-SA/Dx5 tumors. The protocols for animal experimentation described in this paper were previously approved by the Animal Research Committee, Akita University; All subsequent animal experiments adhered to the "Regulation for Animal Experimentation" of the University (Permit Number: a-1-2320). MES-SA/Dx5 cells (5×10^6^ cells in 0.1 ml) were implanted subcutaneously on the left flank. Treatment was initiated in animals when the tumor volume reached >80 mm^3^. Animals weighed 14.2−19.6 g at the beginning of treatment. Intraperitoneal administration of K252a dissolved in physiological saline (1 mg/kg) was performed every 3 days. As controls, vehicle alone or K252b (1 mg/kg) were used. The doses of K252a and K252b chosen for these experiments were based on previous studies [31]. Tumor volumes were measured daily using the formula: tumor volume (mm^3^)  =  length (mm) × [width (mm)]^2^ ×0.5. The tumors were resected after 15 days of treatment, weighed, embedded and sectioned. In addition to hematoxylin and eosin (H&E) staining to assess morphology, *in vivo* cell proliferation and apoptosis were evaluated by Ki-67 immunostaining and the TUNEL assay, respectively, as described [35]. Caspase-3/7 activities in excised tumor samples were measured using the Caspase-3/7 assay (Promega). The tumor tissues were homogenized in extraction buffer: 25 mM HEPES pH 7.5, 5 mM MgCl_2_, 1 mM EDTA, and a protease inhibitor cocktail (Roche). Data were standardized by protein concentration and represented as fold increases relative to controls.

### Statistical Analysis

The Mann−Whitney U test was performed to compare the levels of TrkB and its ligands in human uterine sarcoma cell lines. The effects of drug treatment on tumor volume were analyzed using a Students’ *t*-test. One-way ANOVA, followed by Fisher’s protected least significant difference test, was used to evaluate other differences. Data are expressed as the mean ± SEM.

## Results

### Expression of Trk Receptors and Neurotrophins in Uterine Sarcoma Cells

The expression of Trk receptors and their ligands in uterine sarcoma cells was examined by PCR and immunostaining. Transcripts encoding TrkB, p75NTR, BDNF and NT4/5 were detected in both MES-SA and MES-SA/Dx5 cells (Fig. 1A). Other Trk receptor family members (TrkA and TrkC) and their ligands (NGF and NT3) were also expressed in both cell lines (Figure S1). In leiomyosarcoma cell line, SKN, expression of TrkB, p75NTR, BDNF and NT4/5 were detected, too (Fig. S2A). The Trk receptors and ligands were also detected in human uterine leiomyosarcoma tissue by RT-PCR (Fig. 1B). Quantitative real-time RT-PCR indicated that the levels of TrkB, BDNF and NT4/5 transcripts were higher in MES-SA/Dx5 cells than in MES-SA cells (Fig. 1C). The expression of TrkB, BDNF and NT4/5 in uterine sarcoma cells was confirmed at the protein level using immunofluorescent staining (Fig. 1D; MES-SA/Dx5 cells).

### Increases in TrkB and BDNF and Decreases in a Downstream Effector of TrkB, Galectin-1 in Human Uterine Leiomyosarcoma

In human uterine samples, the expression levels of TrkB and its ligands were compared among uterine myometrium (n = 8), leiomyoma (n = 8) and leiomyosarcoma (n = 7) by quantitative real-time RT-PCR. The levels of TrkB and BDNF transcripts were significantly increased in uterine leiomyosarcoma as compared to those in myometrium and leiomyoma (Fig. 2A and B), whereas NT4/5 expression was insignificant among the tissues (Fig. 2C). In uterine leiomyosarcoma group, three patients with high TrkB transcript levels also showed increased BDNF expressions (Fig. 2A and B, closed circles). To examine the downstream effectors of TrkB signaling, we also evaluated galectin-1 expressions. Quantitative real-time RT-PCR revealed that galectin-1 levels were significantly high in leiomyoma, but low in uterine leiomyosarcoma (Fig. 2D).

### Effect of *in vitro* Suppression of TrkB Receptors on Uterine Sarcoma Cell Growth

To determine the roles of endogenous TrkB ligands in cell proliferation and apoptosis of sarcoma cells, MES-SA and MES-SA/Dx5 cells, were incubated with K252a or the TrkB ectodomain. Cell proliferation was inhibited by treatment with either the K252a or TrkB ectodomain, but not with the K252b (Fig. 3A). In leiomyosarcoma cells, SKN, also presented same inhibition patterns (Fig. S2B). Furthermore, we found that apoptosis, as measured by caspase-3/7 activities and the proportion of TUNEL-positive cells, increased after treatment with K252a (Fig. 3B and C, Fig. S2C).

### Effects of BDNF Treatment on Uterine Sarcoma Cell Proliferation *in vitro*

To examine the effect of BDNF on cell proliferation, cells were cultured with or without different doses of BDNF. As shown in Figure 4, BDNF treatment increased cell proliferation by 20% in MES-SA/Dx5 cells, whereas changes in MES-SA cells were insignificant. At high density (1,000 cells/well), treatment with exogeneous BDNF had no effect on the proliferation of either cell line (data not shown).

### *In vivo* Effects of a Trk Receptor Inhibitor on Uterine Sarcoma Growth

To determine whether the observed *in vitro* inhibition of sarcoma cell growth by Trk receptor inhibitors could be translated into *in vivo* antitumor activity, K252a was administered to athymic nude mice bearing tumor xenografts of MES-SA/Dx5 cells, which were chosen for these studies due to their high BDNF and TrkB expression levels and multidrug-resistant character that could be more severe model of uterine sarcoma for *in vivo* analyses. When the tumor volume reached >80 mm^3^, animals were given vehicle alone, K252a, or K252b at 1 mg/kg every 3 days for 15 days. Tumors in animals treated with K252a were significantly smaller than those that received vehicle or K252b (Fig. 5A). No tumor metastasis nor critical side effects were observed during the experimental period. Body weight was not significantly affected by the treatments (vehicle, 18.75±0.49 g; K252a, 17.77±0.69 g; K252b, 18.45±0.50 g).

Excised tumors were further examined to determine the *in vivo* effects of K252a. Histopathological inspection by H&E staining revealed decreases in the mitotic activity and increases in number of tumor cells with chromatin condensation within tumors of K252a-treated mice (Fig. 5B, upper panels), suggesting suppression of cell proliferation and survival. Ki-67 staining confirmed the effects of K252a treatment on the suppression of cell proliferation (Fig. 5B, middle panels), whereas TUNEL labeling indicated increases in apoptosis (Fig. 5B, lower panels). Moreover, we characterized K252a-induced apoptosis in tumors by quantifying caspase activities and observed a 2.2-fold increase in the activation of caspase-3/7 within tumors of K252a-treated mice (Fig. 5C). K252b treatment had no effect as assessed by all of the tested parameters.

## Discussion

Our results showed that the TrkB receptor and its ligands were expressed in uterine leiomyosarcoma, and that TrkB signaling stimulated cell growth in uterine sarcoma cell lines (MES-SA and MES-SA/Dx5 cells) and uterine leiomyosarcoma cell lines (SKN) (Fig. S2). Due to lack of tumorigenesis in athymic nude mice, we have not used the SKN cells for *in vivo* analyses. MES-SA/Dx5 cells were established from MES-SA cells by selecting the cells under doxorubicin treatment [26]. This cell line showed multi-drug resistance against doxorubicin, paclitaxel, vinblastine, vincristine and etoposide using in current chemotherapy of uterine leiomyosarcoma (e.g. 175-fold resistance to paclitaxel compared to MES-SA) [36]. Despite to its multi-drug resistance potential, suppression of TrkB signaling induced the inhibition of cell growth and survival in MES-SA/Dx5 cells, suggesting an important role of endogenous TrkB signaling. The TrkB receptor and its ligands were also expressed in different uterine leiomyosarcoma cell line, SKN (Fig. S2A). Although SKN is derived from a uterine leiomyosarcoma distinct from that which gave rise to the MES-SA and MES-SA/Dx5 cells [37], its proliferation was also suppressed by inhibiting TrkB signaling (Fig. S2B), suggesting important endogenous roles of BDNF/TrkB signaling in growth of leiomyosarcoma cells. BDNF/TrkB-induced cell proliferation has been reported in other cancers, including neuroblastoma [38], Wilms’ tumor [39], and pancreatic adenocarcinoma [40]. In these cancers, increased expression of TrkB and/or BDNF is associated with poor clinical outcomes. Overexpression of BDNF and TrkB is also associated with poor outcomes for neuroblastoma patients [19], [41]. Indeed, the BDNF transcript levels in neuroblastomas with a poor prognosis were 2-fold higher as compared to those in tumors with good outcomes [41]. In this study, we found that both BDNF and TrkB mRNA levels were increased in the multidrug-resistant (MES-SA/Dx5), as compared to the drug sensitive (MES-SA), uterine sarcoma cell line. Consistent with previous studies in neuroblastoma [21], [38], BDNF/TrkB signaling in uterine sarcoma may play a role in the acquisition of drug resistance.

In uterine leiomyosarcoma samples obtained from patients, the levels of BDNF and TrkB were significantly high as compared with uterine myometrium and leiomyoma. Interestingly, the leiomyosarcoma patients with high BDNF expression also showed significantly high levels of TrkB, but not NT4/5. Due to poor outcomes exhibiting overexpression of BDNF and TrkB in neuroblastomas [19], [41], increased levels of BDNF and TrkB are also supposed to reflect the prognosis of leiomyosarcoma. Among possible prognostic factors, execution of surgical treatment together with clinical stage was shown to mostly influence the outcome of this disease [12], [42]. In our leiomyosarcoma cases, clinical stage of the group with high-BDNF/TrkB was stage I (n = 1) and stage IV (n = 2), whereas low-BDNF/TrkB group was stage I (n = 2) and stage IV (n = 2). All stage I patients received surgical resection of tumor. Therefore, future studies on more cases would be requited to evaluate the prognosis of individual patients based on the expression levels of BDNF and TrkB. We further analyzed galectin-1 expression as a candidate for the downstream effector of TrkB signaling in uterine leiomyosarcoma samples.

The expression of galectin-1 is shown to regulate by the activation of TrkB in neuroblastoma cells [43] and involved in modulating cellular proliferation, survival and migration of malignant cells [44]. In uterine leiomyosarcomas, galectin-1 levels were significantly low, consistent with earlier reports showing that galectin-1 staining was high in leiomyoma, but weak in leiomyosarcoma and myometrium [45]. Because treatment of human sarcoma cells, SK-UT-1 and SK-LMS-1 with galectin-1 inhibited cell proliferation [46], low levels of galectin-1 in uterine leiomyosarcoma may contribute to the tumor growth. Future studies of galectin-1 in uterine leiomyosarcoma would provide a better understanding of BDNF-TrkB signaling mediated-cell growth.

Although NT4/5 is another TrkB ligand, its expression levels in uterine sarcoma cells are low, suggesting that BDNF acts as the predominant ligand in the context of uterine sarcoma cell growth. Similar expression patterns were demonstrated in choriocarcinoma cells [31], whereas both BDNF and NT4/5 contributed to cellular survival of breast cancer [47]. In our study, stimulation of cell proliferation by exogenous BDNF was observed in MES-SA/Dx5, but not MES-SA, cells. Because the expression of TrkB in MES-SA cells was low in contrast to MES-SA/Dx5 cells, one possibility is that the receptors in the former cell line were already saturated by endogenous BDNF and thus unable to mount an additional response in the presence of exogenous BDNF. The effectiveness of endogenous BDNF in MES-SA/Dx5 cell proliferation was relatively low, and stimulation with exogenous BDNF was not evident when cells were cultured at high density, suggesting that the concentration of endogenous BDNF in culture medium could be increased under high-density conditions.

We demonstrated the ability of endogenous BDNF to promote the proliferation and survival of uterine sarcoma cells using the TrkB ectodomain and the Trk inhibitor, K252a. Because the inhibitory effect of K252a on cell proliferation was similar to that of the TrkB ectodomain *in vitro*, K252a is likely to be specific for TrkB, although other members of the Trk family, including TrkA and TrkC, were expressed in these sarcoma cells (Fig. S1).

The expression of both TrkB and TrkB ligands in uterine sarcoma cells suggested the presence of autocrine loop of TrkB signaling. However, *in vivo* model, endocrine actions of other tissues-derived mouse BDNF in supporting tumor cell growth cannot be ruled out. The suppressive effect of K252a on MES-SA/Dx5 tumor growth in athymic nude mice indicated that BDNF/TrkB signaling also played a significant role in uterine sarcoma growth and protection against apoptosis *in vivo*. K252a is a small molecule (MW = 467 Da), easily delivered to tumor xenografts via i.p. administration with no overt side effects. By contrast, the TrkB ectodomain is a large glycosylated protein (MW = 75–100 kDa), which is unstable in the body. Thus, we used K252a for *in vivo* studies. Although uterine sarcoma is a highly invasive and metastatic cancer, MES-SA tumor cells xenografted into mouse uterine muscle failed to metastasize after 42 days [48]. Similarly, we found no metastatic lesions up to 30 days.

Several molecular targeted agents have been developed and introduced into clinical trials for the treatment of uterine leiomyosarcoma [49]. Thalidomide, an anti-angiogenic agent, has been investigated for use against uterine leiomyosarcoma [50]. However, it was not effective, showing only 1.9 months of median progression free survival. Clinical trials of bevacizumab, a monoclonal antibody against vascular endothelial growth factor combined with doxorubicine, was conducted for soft tissue sarcomas, including uterine leiomyosarcoma [51]. Although 65% of the patients had stable disease, this regimen was not encouraged due to cardiac toxicity. Accumulating evidence indicates that receptor tyrosine kinases promote tumorigenesis in a number of settings, and these proteins also represent therapeutic targets [52]. Sunitinib malate, a multi-targeted inhibitor of receptor tyrosine kinases, but not Trk family members, was tested against uterine leiomyosarcoma [53]. However, treatment with sunitinib malate was less effective, with only 4 of 23 patients (17.4%) that survived progression free up to 6 months. Therefore, the development of new, effective molecular-targeted agents for uterine leiomyosarcoma is anticipated.

In conclusion, our findings demonstrated important roles for TrkB signaling in uterine leiomyosarcomas based on *in vitro* and *in vivo* studies. Moreover, we demonstrated the expression level of TrkB and BDNF in uterine leiomyosarcoma associated with clinical malignancy in patients. Further elucidation of the mechanisms of BDNF/TrkB-induced leiomyosarcoma growth could lead to novel therapeutic approaches for the treatment of patients with this disease.

## Supporting Information

Figure S1 **Expression of TrkA, TrkC, NGF and NT3 in uterine sarcoma cell lines.** Expression of TrkA, TrkC, NGF and NT3 mRNA was detected by RT-PCR in the uterine sarcoma cell lines, MES-SA and MES-SA/Dx5. As loading controls, β-actin mRNA levels were assessed. The negative controls lacked template DNA.
(TIF)

Figure S2 **Expression of TrkB, p75NTR and their ligands in uterine leiomyosarcoma cell line, SKN and roles of endogenous TrkB signaling in** ***in vitro*** **cell proliferation and survival** (**A**) Expression of TrkB, p75NTR and their ligands mRNA was detected by RT-PCR. The levels of β-actin mRNA were assessed as loading internal controls. The negative controls lacked template DNA. nc, negative control. (**B**) Effects of suppression of endogenous TrkB signaling on cell proliferation. SKN cells were cultured in medium alone (control, C), or with different doses of the TrkB ectodomain (TrkB EC), the pan-Trk inhibitor, K252a, or its inactive analogue, K252b. Cell proliferation activity was determined using the cell proliferation reagent WST1 (n = 6). *Columns*, mean; *bars*, SE. *, *P*<0.05 vs. control. (**C**) Effects of suppression of endogenous TrkB signaling on cell survival. SKN cells were treated without or with K252a (1 µM). Apoptosis was determined using the caspase-3/7 assay (n = 6). Data were represented as fold increases relative to controls at individual culture times. *Columns*, mean; *bars*, SE. *, *P*<0.05 vs. control.
(TIF)
