# Supplementary figures and images for: Inhibition of Uterine Sarcoma Cell Growth through Suppression of Endogenous Tyrosine Kinase B Signaling
Source: PLoS One. 2012 Jul 23;7(7):e41049. doi: 10.1371/journal.pone.0041049 (PMC3402458; doi:10.1371/journal.pone.0041049)

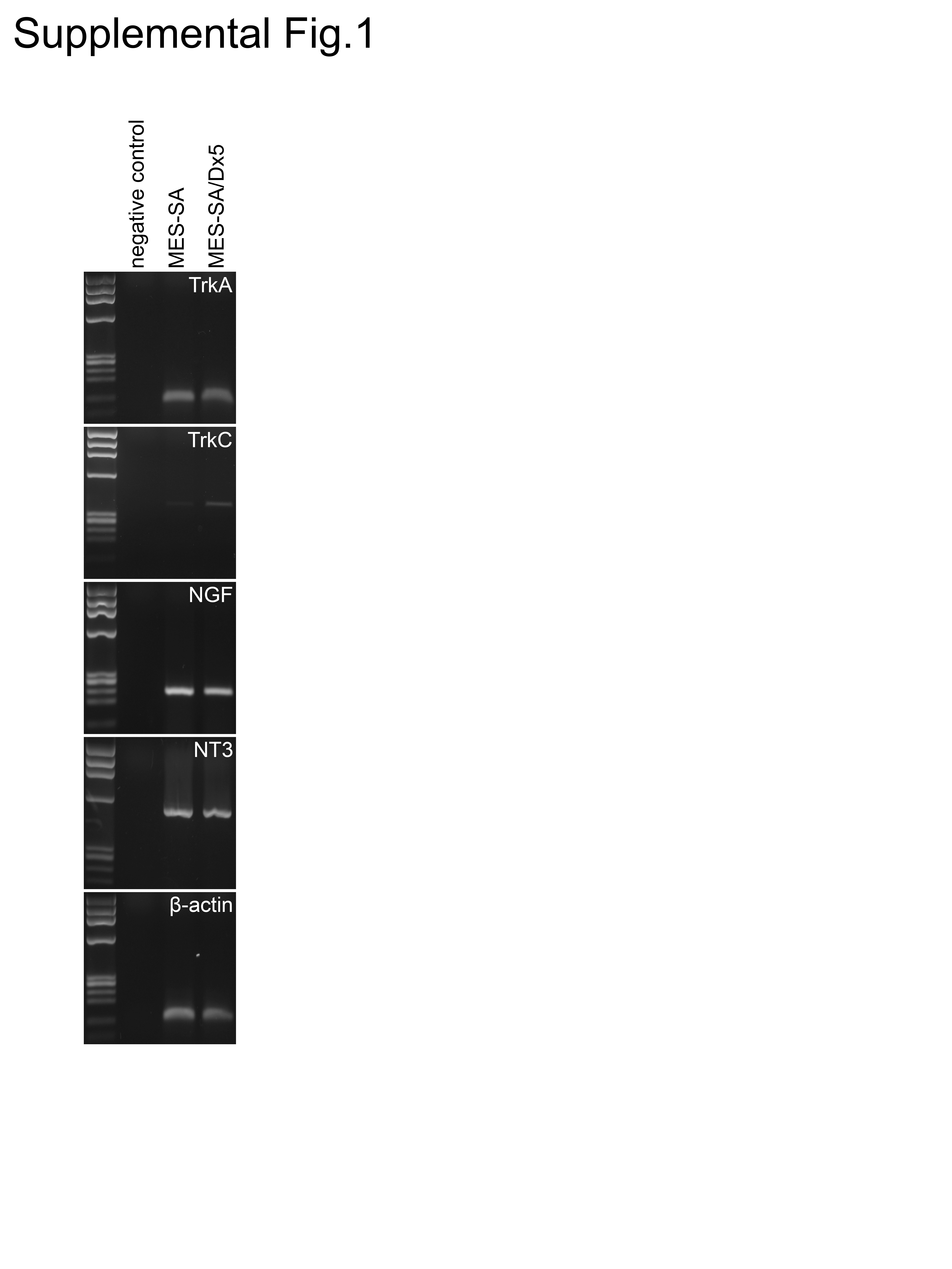

Supplement: Figure S1 — Expression of TrkA, TrkC, NGF and NT3 in uterine sarcoma cell lines. Expression of TrkA, TrkC, NGF and NT3 mRNA was detected by RT-PCR in the uterine sarcoma cell lines, MES-SA and MES-SA/Dx5. As loading controls, β-actin mRNA levels were assessed. The negative controls lacked template DNA. (TIF) [file pone.0041049.s001.tif]

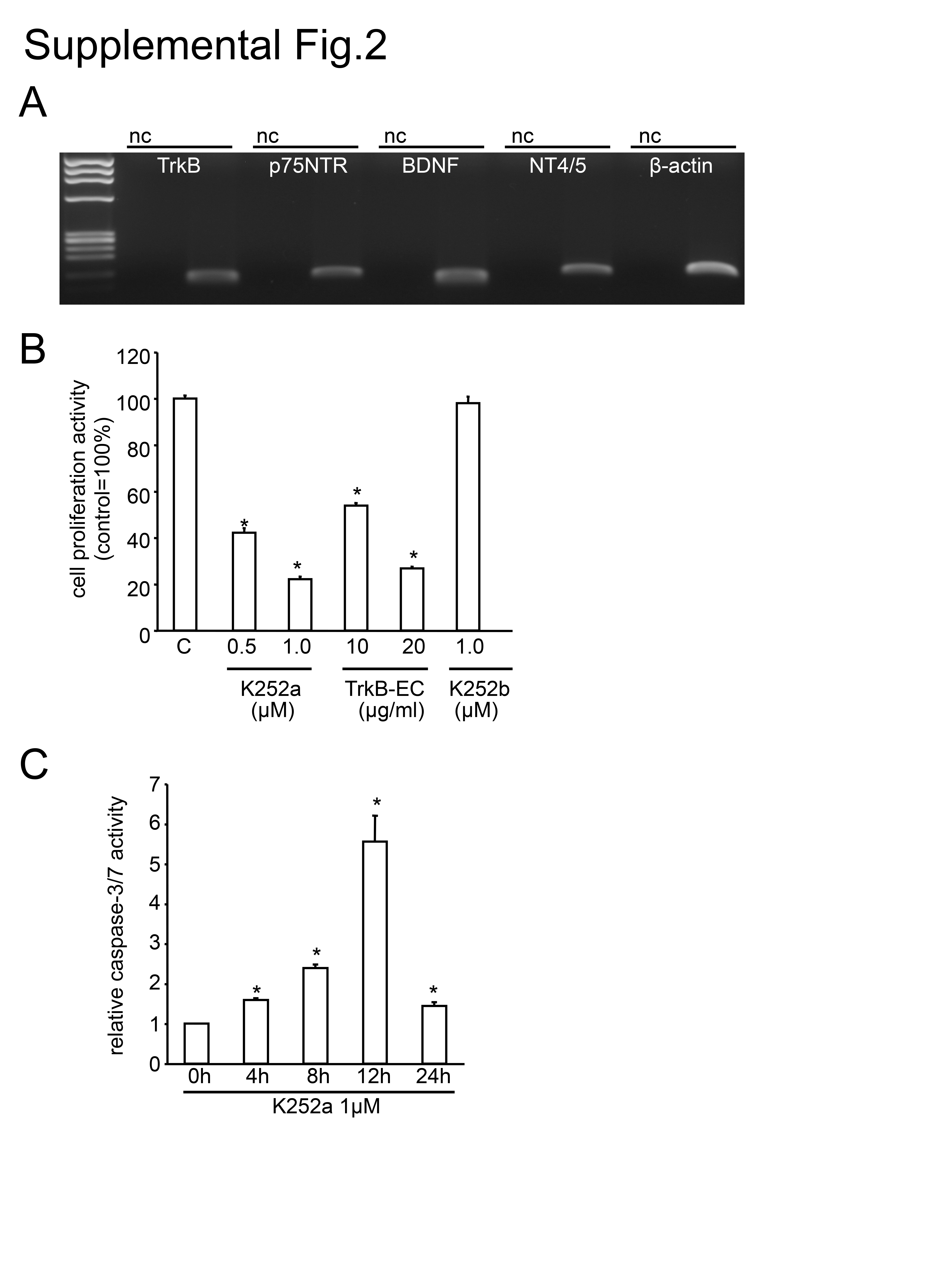

Supplement: Figure S2 — Expression of TrkB, p75NTR and their ligands in uterine leiomyosarcoma cell line, SKN and roles of endogenous TrkB signaling in in vitro cell proliferation and survival (A) Expression of TrkB, p75NTR and their ligands mRNA was detected by RT-PCR. The levels of β-actin mRNA were assessed as loading internal controls. The negative controls lacked template DNA. nc, negative control. (B) Effects of suppression of endogenous TrkB signaling on cell proliferation. SKN cells were cultured in medium alone (control, C), or with different doses of the TrkB ectodomain (TrkB EC), the pan-Trk inhibitor, K252a, or its inactive analogue, K252b. Cell proliferation activity was determined using the cell proliferation reagent WST1 (n = 6). Columns, mean; bars, SE. *, P<0.05 vs. control. (C) Effects of suppression of endogenous TrkB signaling on cell survival. SKN cells were treated without or with K252a (1 µM). Apoptosis was determined using the caspase-3/7 assay (n = 6). Data were represented as fold increases relative to controls at individual culture times. Columns, mean; bars, SE. *, P<0.05 vs. control. (TIF) [file pone.0041049.s002.tif]
